# Genetic Performance of the Semidwarfing Allele* sd1* Derived from a Japonica Rice Cultivar and Minimum Requirements to Detect Its Single-Nucleotide Polymorphism by MiSeq Whole-Genome Sequencing

**DOI:** 10.1155/2018/4241725

**Published:** 2018-04-03

**Authors:** Motonori Tomita, Kazuo Ishii

**Affiliations:** ^1^Research Institute of Green Science and Technology, Shizuoka University, Shizuoka 422-8529, Japan; ^2^Faculty of Agriculture, Tokyo University of Agriculture and Technology, Fuchu, Tokyo 183-8538, Japan

## Abstract

The influence of the semidwarfing gene* sd1* derived from the rice cultivar Jukkoku (Jukkoku*_sd1*) and IR8 (IR8*_sd1*), which contributed to the Green Revolution,* d60* from Hokuriku 100, as well as the combination of* sd1* and* d60* (Jukkoku*_sd1 *plus* d60* and IR8*_sd1* plus* d60*), was investigated using isogenic lines raised by backcrossing with the cultivar Koshihikari. The isogenic lines carrying Jukkoku*_sd1*, IR8*_sd1*,* d60*, Jukkoku*_sd1 *plus* d60*, and IR8*_sd1* plus* d60* had considerably shorter culm lengths than Koshihikari by 19.2%, 22.8%, 26.0%, 45.1%, and 43.4%, respectively. The* sd1 *plus* d60* lines showed additively reduced culms, indicating that the function of* d60* was different from* sd1*. In contrast to the culm reduction, Jukkoku*_sd1* showed productive merit with a panicle length of 2.5% greater than the origin. MiSeq next-generation sequencer was used to optimize a minimum scale to detect Jukkoku*_sd1* in practical breeding. Mapping with the reference genome of Nipponbare gained the average depths of Koshihikari Jukkoku_*sd1* and Koshihikari being 9.17 and 7.29, respectively. Comparing the vcf files of the entire genomes of Koshihikari Jukkoku_*sd1* and the virtual Koshihikari revealed a G to T SNP at position 38,382,746 in the* sd1 *locus on chromosome 1 of Koshihikari, causing a loss-of-function mutation of GA20-oxidase.

## 1. Introduction

Semidwarfism of rice improves phenotype (light-interception properties and harvest index), nitrogen responsiveness, and lodging resistance and is thus an important trait worldwide. The International Rice Research Institute produced a cross between Peta, a tall* indica* variety (culm length, 150–180 cm), characterized by abundant, long hanging leaves, commonly grown in tropical Asia, and Dee-geo-woo-gen (DGWG), a Taiwanese indigenous semidwarf variety, to improve its lodging resistance and light-interception properties. The resulting semidwarf rice variety IR8 (culm length, 90–100 cm), developed in 1966, has dramatically improved rice yields and brought the Green Revolution to tropical Asia [1].

The semidwarf trait has also been introduced into rice cultivars grown in other countries. In Japan, Hoyoku, Shiranui, and Reihou, which are representative main cultivars grown in the Kyushu region, were developed using the indigenous semidwarf cultivar Jukkoku [2]. A series of cultivars, such as Akihikari and Niigata Wase, were developed in the 1970s in the Tohoku region using the semidwarf cultivar Reimei, which was induced by gamma-ray irradiation of Fujiminori [3]. In the USA, Calrose 76 (culm length, ~90 cm) was developed in 1976 also by gamma-ray irradiation of the* japonica* variety Calrose (culm length, ~120 cm) [4–6]. In Korea,* d47* derived from IR8 was also introduced into Tongil by japonica-indica hybridization [7].

Advances in genetic research have facilitated the identification of genes responsible for semidwarfism in rice. The incomplete recessive gene* d47* is responsible for the semidwarfism of DGWG, the parent line of IR8 [8–10]. Later, the incomplete recessive gene* sd1* in Calrose 76 was shown to be allelic to* d47* [4, 11, 12]. Moreover, allelic relationships of the semidwarf gene have been found in Taichung Native 1, derived from DGWG; Shiranui, derived from Jukkoku; and* d49* in the mutant cultivar Reimei [13, 14]. Finally, all these semidwarf cultivars carry alleles at the* sd1* locus, despite their different parentage [14–18]. The* sd1* alleles, on the long arm of chromosome 1, encode loss-of-function mutations in GA20-oxidase (*OsGA20ox2*), which regulates the synthesis of biologically active gibberellins (GAs), which catalyze three steps in the GA biosynthesis pathway [15–18]. Taken together, a single semidwarf gene,* sd1*, solely confers the semidwarf phenotype of cultivars commonly grown around the world due to its nondetrimental effects on grain yield [19].

This narrow genetic base of current semidwarf rice cultivars has led to reduced genetic diversity in rice [20–22]. Thus, it is necessary to identify a novel semidwarf gene alternative to* sd1* and to utilize it to extend genetic diversity in semidwarf cultivars. A novel dwarf gene,* d60*, which was found in the semidwarf mutant Hokuriku 100, developed by exposing Koshihikari to 20 kR of gamma radiation [23, 24], is thus of particular importance. Unlike* sd1*, which is inherited as a single recessive gene,* d60* complements the gametic lethal gene* gal*, which is carried by many rice varieties. Consequently, the cross between Hokuriku 100 and Koshihikari exhibits a unique genotype ratio of 4*D60D60* : 4*D60d60* : 1*d60d60* [23–25].

Although many semidwarf genes allelic to* sd1* have been identified in different cultivars (DGWG, Jukkoku, Reimei, and Calrose 76), differences in their influences have not been elucidated. Thus, investigating the differences in ecological and phenotypic traits in relation to yield between* d60*- and* sd1*-carrying plants, as well as those among* sd1* allelic variants of different origins, will be beneficial for future use of* d60* and multiple allelic variants of* sd1*. The first author developed isogenic semidwarf lines by continuously backcrossing* sd1* of Jukkoku (Jukkoku_*sd1*) and* sd1* of Kinuhikari (IR8_*sd1*) and* d60* and both genes into the Koshihikari background and maintained the lines to investigate the resulting traits. Hence, the study was conducted with the following specific objectives: (a) investigate and quantify the differences between* d60*- and* sd1*-carrying NILs, in relation to yield, to assess the utility of* sd1* and* d60* semidwarf genes and (b) sequence NILs by MiSeq to detect the differences between* Sd1*/*sd1* target sites.

## 2. Materials and Methods

### 2.1. Plant Genetic Materials

**Figure 1:**
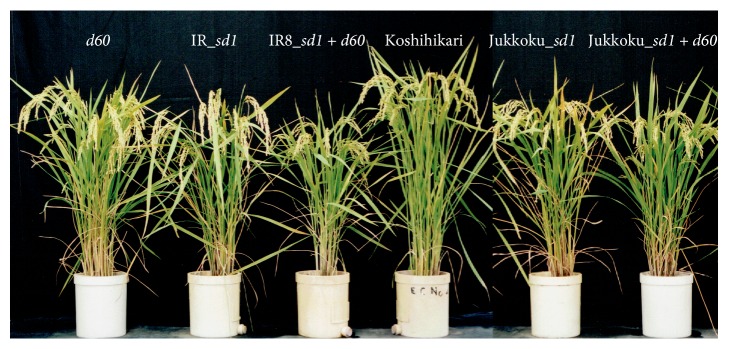
Semidwarf isogenic lines with the genetic background of Koshihikari, which contained* d60*, IR_*sd1*, IR8_*sd1* +* d60*, Jukkoku_*sd1*, and Jukkoku_*sd1* +* d60*, respectively.

As shown in Figure 1, the following rice cultivars were examined: Koshihikari, Koshihikari Jukkoku*_sd1* (Koshihikari*∗*6//Koshihikari/Jukkoku B_6_F_4_), Koshihikari IR8*_sd1* (Koshihikari*∗*3//Koshihikari/Kinuhikari B_3_F_3_, Kinuhikari carrying* sd1* of IR8 [26]), Koshihikari* d60 *(Koshihikari*∗*3//Koshihikari/Hokuriku 100 B_3_F_3_), Koshihikari Jukkoku*_sd1* plus* d60* (Koshihikari Jukkoku*_sd1*/Koshihikari* d60*), and Koshihikari IR8*_sd1* plus* d60* (Koshihikari IR8*_sd1*/Koshihikari* d60*). In each backcross experiment, short-culm BC_*n*_F_2_ plants were backcrossed with Koshihikari as the recurrent female parent in each generation. Each semidwarf phenotype was fixed by the BC_*n*_F_3_ generation. The percentage of genetic materials from Koshihikari in the isogenic semidwarf lines was calculated as follows: For Koshihikari carrying Jukkoku_*sd1* (B_6_F_4_), (1 − (1/2)^6^) × 100 ≈ 98.4%. For B_3_F_3_, (1 − (1/2)^3^) × 100 = 87.5%.

### 2.2. Cultivation

Rice seeds were collected from stocks kept in a refrigerator. Seeds of each line were immersed in just enough water to cover them. Water was changed every day for 7 days (May 2 to May 8) during germination stimulation. Seedlings were grown in special boxes (30 × 15 × 3 cm) for approximately 20 days in a greenhouse: two seeds were planted in each cell (2 × 2 × 3 cm) in the box on May 11 and 12 and watered twice a day (07:00 and 12:00). Seedlings were then individually transplanted into a paddy field (120 m^2^: 6.0 × 20.0 m) of the University Farm on June 8. The paddy field received 4.0 kg of basal fertilizer containing nitrogen, phosphorus, and potassium (weight ratio, nitrogen : phosphorus : potassium = 2.6 : 3.2 : 2.6). Except for the period of mid-season drainage (July 10 to July 17), the water level was maintained at 5–7 cm above ground to prevent seedlings from submersing. A herbicide (Joystar L floable) was applied on June 20 to kill weeds growing uncontrollably and the water was then kept at a high enough level to cover the weeds for 1 week. Koshihikari, Koshihikari carrying Jukkoku_*sd1*, Koshihikari carrying IR8_*sd1*, Koshihikari carrying* d60*, Koshihikari carrying* d60* and Jukkoku_*sd1*, and Koshihikari carrying* d60* and IR8_*sd1* were grown in 4 m^2^ plots (2 × 2 m) (two instances per line). Basal fertilizer was applied to give 0.43 g/m^2^ nitrogen, 0.53 g/m^2^ phosphorus, and 0.53 g/m^2^ potassium. After ripening, 10 plants per genotype were sampled twice to assess the following traits: panicle heading time, culm length, internode interval, and panicle length.

### 2.3. Investigation of Agronomic Traits

The time when the tip of the panicle first emerged from the flag leaf sheath was recorded as the heading time for all plants. Ten plants typical of each line were selected. The sampling procedure was performed twice. Sampled plants were air-dried and the following phenotypic traits were assessed or measured: (1) culm length (the length between the ground surface and the panicle end of the main culm), (2) internode length (the lengths between two neighboring internodes of the upper five internodes), and (3) panicle length (the length between the panicle base and the tip of the panicle). Differences in phenotypic traits between each line carrying a semidwarf gene (or genes) and Koshihikari were obtained using the following equation: percent difference = [(measurements of each line) − (measurements of Koshihikari)]/(measurements of Koshihikari) × 100.

### 2.4. Next-Generation Sequencing (NGS) Analysis

An advantage of genomics is the development of a next-generation sequencer that can decode DNA sequences at the giga level. Development of the next-generation DNA sequencer was advanced under the Affordable Care Act aims to realize societal implementation of medical genomics [27, 28]. The desktop-type next-generation sequencer MiSeq has the ability to read 15 million DNA sequences in one run. Generally, whole-genome sequencing analysis required 30 times of the given genome size (30× genome coverage). According to this standard scale, the MiSeq treats only one rice sample per run, because the 15 million DNA amount is just enough to reconstruct a single rice genome. However, it is a big problem that the running cost is excessively high to use the MiSeq at the practical breeding to detect target genes. So, in this study, to achieve a minimum scale to detect target genes with a reasonable cost, we try to detect Jukkoku_*sd1* at only 5× rice genome coverage by using the MiSeq. The semidwarf gene* sd1* (a loss-of-function mutation of the GA20-oxidase encoding gene) was transferred to Koshihikari by consecutive backcrosses to prepare a semidwarf Koshihikari named Koshihikari Jukkoku_*sd1*. The Koshihikari Jukkoku_*sd1* backcross was used to detect single-nucleotide polymorphisms (SNPs) in Jukkoku-derived* sd1* by NGS. Whole-genome analysis was conducted using Koshihikari Jukkoku_*sd1* and Koshihikari. Genomic DNA was extracted from each cultivar by the CTAB (hexadecyltrimethylammonium bromide) method. Genomic DNA was tagged and fragmented to an average length of 500 bp using the Nextera® transposome-based approach. After purification of the transposome with DNA Clean & Concentrator™-5 (Zymo Research, Irvine, CA, USA), adaptors for fixation on the flow cell were synthesized at both ends of each fragment using a polymerase chain reaction (PCR). Then, the DNA fragments were subjected to size selection using AMPure XP magnetic beads (Beckman Coulter, Inc., Brea, CA, USA). Finally, qualitative and quantitative measurements were performed using a Fragment Analyzer™ (Advanced Analytical Technologies, Inc., Ankeny, IA, USA) and a Qubit® 2.0 Fluorometer (Thermo Fisher Scientific, Waltham, MA, USA) to prepare a DNA library for NGS. Aiming to achieve 5× rice genome coverage, a MiSeq next-generation sequencer was used to simultaneously analyze five lines; namely, 4-5 ng of five DNA libraries was applied in each MiSeq run. Clusters then were formed on the flow cells by bridge-PCR and each pair-end of a 250 bp read was sequenced. Resulting sequenced reads were mapped using BWA software with the Nipponbare genome [29] as a reference. Then, SNPs and Indels were detected using SAMtools software (http://samtools.sourceforge.net/).

## 3. Results

### 3.1. Effects of Semidwarf Genes* sd1* and* d60* on Heading Time

**Figure 2:**
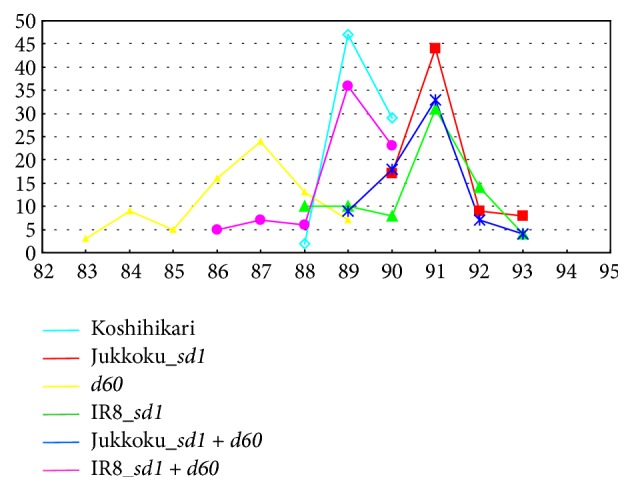
Panicle heading time of isogenic lines introgressed with Jukkoku_*sd1*, IR8_*sd1*,* d60*, and their combinations.

The days to heading were compared (Figure 2). The earliness of varieties of Koshihikari and donors of semidwarf genes is as follows: Koshihikari, an early-medium maturing variety; Jukkoku, a medium-late maturing variety; Kinuhikari, an early-medium maturing variety; and Hokuriku 100, an early-medium maturing variety. The heading panicles were observed first in Koshihikari carrying IR8_*sd1* (83 days to heading) and the latest heading panicles were observed in Koshihikari carrying Jukkoku_*sd1* or Jukkoku_*sd1* plus* d60* (93 days to heading). However, the difference in the average number of days to heading was the largest between lines carrying IR8_*sd1* (86.5 days) and those carrying* d60* (90.5 days), but this 4-day difference was thought to be minor (Figure 2). Thus, it was concluded that the time required for maturing was comparable among lines and the differences in traits, such as panicle length, were attributed to genetic factors.

### 3.2. Effects of Semidwarf Genes* sd1* and* d60* on Traits Related to Plant Type

**Figure 3:**
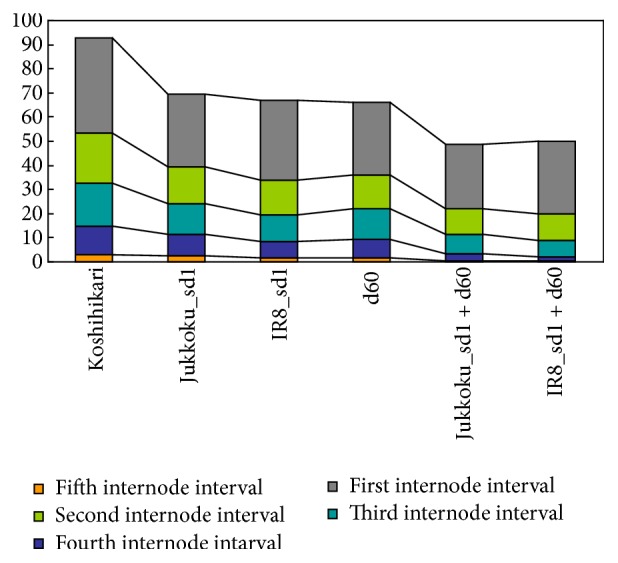
Effects of the semidwarfing genes Jukkoku_*sd1*, IR8_*sd1*, and* d60* and their combinations on internode constitution.

The mean values of the individual traits in plants of each genotype were calculated and the percent differences relative to the corresponding mean values of Koshihikari were compared. Introduction of a semidwarf gene (or genes) resulted in a reduction in culm length. The mean culm length of Koshihikari was 88.8 cm, while those of lines carrying Jukkoku_*sd1*, IR8_*sd1*,* d60*, Jukkoku_*sd1* plus* d60*, and IR8_*sd1* plus* d60* were 71.8, 68.5, 65.7, 48.6, and 50.2 cm, respectively (Table 1 and Figure 3). Similarly, internode intervals were also reduced by the introduction of at least one semidwarf gene. While reductions in internode intervals were relatively uniform in lines carrying Jukkoku_*sd1* (23.9%, 27.5%, 27.0%, 23.3%, and 19.4%: percent difference, from upper to lower internode intervals), the reduction in length between the third and fourth and between the fourth and fifth internodes was markedly large in lines carrying IR8_*sd1* (16.4%, 31.5%, 37.0%, 42.6%, and 45.0%: percent difference, from upper to lower internode intervals). Reductions in internode intervals were relatively uniform in lines carrying* d60* (24.2%, 32.6%, 27.4%, 34.5%, and 45.7%: percent difference, from upper to lower internode intervals), while percent differences were larger than those observed in lines carrying Jukkoku_*sd1*. In the* sd1* plus* d60* lines, marked reductions were observed in length between neighboring internodes, probably attributed to the additive effect of* sd1* and* d60* (Table 1 and Figure 3). The interval between the fourth and fifth internodes often disappeared in plants carrying IR8-*sd1* and in* sd1* plus* d60* lines, which showed a marked reduction in length between the lower internodes.

**Figure 4:**
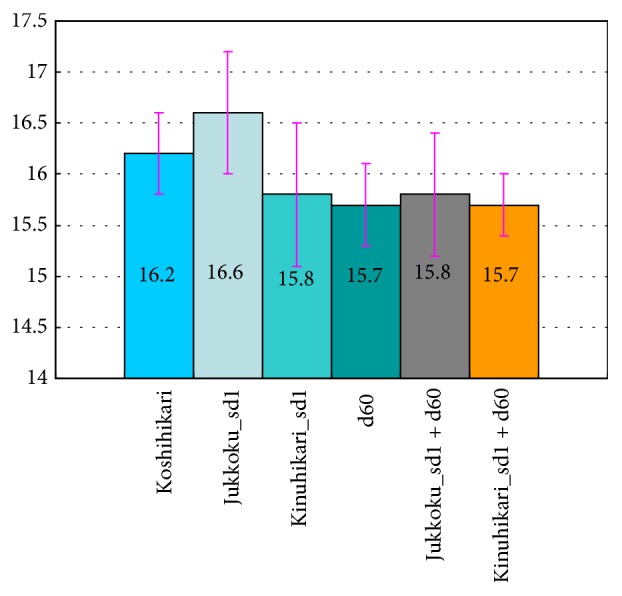
Effects of the semidwarfing genes Jukkoku_*sd1*, IR8_*sd1*, and* d60* and their combinations on panicle length.

In contrast, as shown in Figure 4, the effect of a semidwarf gene (or genes) on panicle length (−3.0 to +2.5%), which was smaller than that for internode intervals (−16 to −92%), was negligible in practical agriculture. Particularly, the mean length of panicles in the line carrying Jukkoku_*sd1* was 16.2 cm, which was solely 2.5% longer than that of Koshihikari. The panicle length of the line carrying Jukkoku_*sd1* was longer than that of the original cultivar Koshihikari: not only the mean value but also the value including the standard deviation caused by circumstance. Therefore, there was certain merit in increasing the production of Jukkoku_*sd1* as compared to other semidwarf genes.

### 3.3. NGS Analysis to Detect Jukkoku-Derived* sd1* with a Minimum Scale

**Figure 5:**
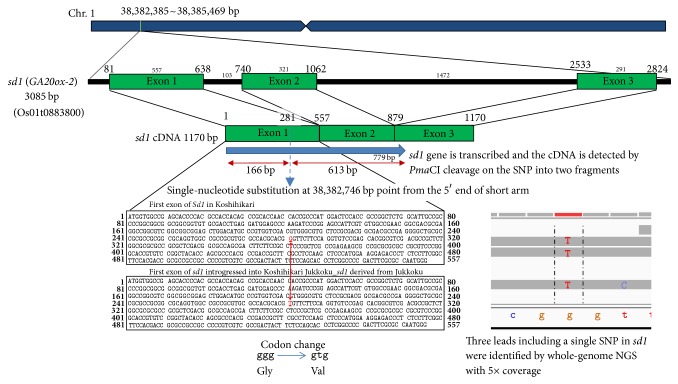
SNP in Jukkoku-derived* sd1* identified by whole genomic NGS for the genome of Koshihikari Jukkoku_*sd1* with coverage 5×.

Using the MiSeq sequencer, we obtained a total read length of 2.79 × 10^9^ bp from the total read number of 9.53 × 10^6^ in Koshihikari Jukkoku_*sd1*, while a total read length of 1.92 × 10^9^ bp was obtained from a total read number of 6.13 × 10^6^ in Koshihikari. By mapping the read sequences obtained by NGS using Nihonbare genomic DNA as a reference, sequence coverage rates of 93.5% and 88.5% were attained for Koshihikari Jukkoku_*sd1* and Koshihikari, respectively, while average depths were 9.17 and 7.29, respectively. Furthermore, vcf files of the entire genomes were prepared and the whole-genome sequence of Koshihikari Jukkoku_*sd1* was compared with that of the virtual Koshihikari genome. As a result, three reads were obtained, including Jukkoku_*sd1* from Koshihikari Jukkoku_*sd1*, while three reads of* Sd1* were obtained from Koshihikari. The* Sd1*/*sd1* locus (Os01t0883800-01) was localized at positions 38,382,385–38,385,469 from the end of the short arm of chromosome 1 in the Koshihikari genome. The difference observed between the Koshihikari_*Sd1*/Jukkoku_*sd1* alleles was only one SNP from G to T in exon 1 of the GA20-oxidase gene (Figure 5), as reported by Sasaki et al. [17]. In this study, the G to T SNP of the defective GA20-oxidase gene was localized at position 38,382,746 from the end of the short arm of chromosome 1 of Koshihikari. Using this scale of the NGS approach with a coverage of only 5×, the targeted SNP in Jukkoku_*sd1* was successfully identified with three reads, with a cost reduced to 1/5 that of the ordinal whole-genome sequencing with a coverage of 30×.

## 4. Discussion

Environmental degradation caused by global warming, postearthquake salt damage, and radioactive contamination and globalization of agricultural markets due to the Trans-Pacific Partnership are serious issues that call for the innovation of new cultivars to overcome the shortcomings of current crops. The results of this study showed that all tested semidwarf lines had shorter culm lengths than Koshihikari, indicating improved lodging resistance. Furthermore, the leaves were straighter (pointing upwards) in the semidwarf lines than in Koshihikari (Figure 1), indicating improved light-interception properties attributed to the introduction of semidwarf gene(s). Panicle length was solely longer by 2.5% in Koshihikari carrying Jukkoku_*sd1* and shorter in lines carrying IR8_*sd1* by 2.4% or* d60* by 3.0%, as compared with the original cultivar Koshihikari (Table 1 and Figure 4). However, the reduction in panicle length was considerably less than that in culm length (Table 1 and Figure 3; a 22.8% decrease in lines carrying IR8_*sd1* versus a 26.1% decrease in lines carrying* d60*), suggesting that the negative effects of the semidwarf genes* sd1* and* d60* on panicle length were negligible (Table 1 and Figure 4). Ogi et al. [30] and Murai et al. [31] reported that an isogenic line carrying* sd1* was derived from DGWG, which was constructed with the genetic background of Norin 29 and Shiokari, respectively. However, IR8-derived* sd1* did not show effects of increasing panicle length, which was also observed with the genetic background of Koshihikari in this study. On the other hand, the results showed that Jukkoku-derived* sd1* solely increased panicle length by more than 2.5%, as compared to the original cultivar Koshihikari and other semidwarfing alleles.

The first author reported transcription of the* sd1* gene derived from Jukkoku [32]. RT-PCR of root RNA showed that the 779 bp fragment derived from the* sd1* locus was clearly cleaved into 613 bp and 166 bp fragments by* Pma*CI digestion on the SNP in Jukkoku_*sd1*, but there was no cleavage of the* Sd1* locus in Koshihikari (Figure 5). This is the first evidence of the transcription of* sd1*, a defective gene of* GA20ox-2*. Accordingly, Jukkoku_*sd1*, which is substituted by only a single nucleotide against the 385 bp-deficient IR_*sd1* [17] and is actively transcribed, has no deteriorative effect on the panicle and may have a positive effect on panicle elongation by the overflow of nutrition due to culm reduction.

In Japan, Koshihikari suffers considerable lodging damage as a result of frequent powerful typhoons and, thus, the development of lodging-resistant cultivars has been a long-standing challenge. The first author transferred the semidwarf gene Jukkoku_*sd1* to Koshihikari to develop a semidwarf form of Koshihikari which could withstand a typhoon by backcrossing with Koshihikari eight times [33], with more than 99.8% of the background of the Koshihikari genome, except for Jukkoku_*sd1* [32]. This cultivar, which was about 20 cm shorter than Koshihikari, was named Hikarishinseiki (rice cultivar number 12273) [33, 34]. Hikarishinseiki is the first cultivar to be a Koshihikari-type semidwarf with* sd1* registered in Japan and USA [35, 36].

In this study, a MiSeq next-generation sequencer was used to achieve 5× rice genome coverage; namely, 4-5 ng of five DNA libraries was applied in one MiSeq run. Using this approach, the targeted gene mutation in Jukkoku_*sd1* was successfully detected as the SNP (G to T) in the defective GA20-oxidase gene, which had reduced the culm length by a loss-of-function mutation of GA synthesis, localized at position 38,382,746 from the end of the short arm of chromosome 1 of Koshihikari. This would be the minimum scale to detect Jukkoku_*sd1* in practical breeding. In Japan, genetically modified organisms are not acceptable to consumers; thus the target SNP in Jukkoku_*sd1* would be effectively tracked by using NGS of 5× coverage scale in each backcrossed generation with Koshihikari. On the other hand, as the DNA sequence and function of* sd1* have been deciphered [15–18, 37], new breeding methods, such as RNAi gene silencing [38] and genome editing [39], are available in other countries to retard culm length by knockout of* Sd1*.

The effects on phenotypic traits of rice differed between the two semidwarf genes (s*d1* and* d60*) and also between* sd1* loci of different origins (Jukkoku_*sd1* and IR8_*sd1*). The effect on culm length was more pronounced in plants carrying* d60* than in those carrying* sd1* (culm length: Jukkoku_*sd1*, 71.8 cm; IR8_*sd1*, 68.5 cm; and* d60*, 65.7 cm). The reduction in internode intervals was relatively uniform in lines carrying Jukkoku_*sd1* or* d60* (uniform reduction type), while reductions of the third and lower intervals were larger than in the upper intervals in lines carrying IR8_*sd1* (Figure 3 and Table 1). Thus, the center of gravity will be lower in the lower internode reduction type, suggesting higher lodging resistance in lines carrying IR8_*sd1* than in those carrying Jukkoku_*sd1* or* d60*. Although the function of* d60* is not known, it is clearly distinct from that of* sd1*, as the additive double-dwarf effect of* sd1* and* d60* appeared in Jukkoku_*sd1* plus* d60* and IR8_*sd1* plus* d60*, respectively. The results of this study demonstrated that* d60* has a stronger effect than* sd1* on culm length and exerts similar effects on other phenotypic traits as with* sd1*. Although many semidwarf genes are associated with a reduction in panicle length,* d60* does not exert such negative effects on phenotypic traits of rice plants. Taken together, these results showed that* d60* is useful for adding genetic diversity to semidwarf varieties and is thus of particular importance in the field of plant bleeding.

## Figures and Tables

**Table 1:** Effects of the semidwarfing genes Jukkoku_*sd1*, IR8_*sd1*, and *d60* and their combinations on panicle length, culm length, and internode interval.

Gene	Panicle length (cm)	Culm length (cm)	1st internode interval (cm)	2nd internode interval (cm)	3rd internode interval (cm)	4th internode interval (cm)	5th internode interval (cm)
Koshihikari	16.2	88.8	39.7	20.7	17.8	11.6	3.1
Jukkoku*_sd1*	16.6 (+2.5%)	71.8 (−19.2%)	30.2 (−23.8%)	15.0 (−27.5%)	13.0 (−27.0%)	8.8 (−23.3%)	2.5 (−19.4%)
IR8*_sd1*	15.8 (−2.4%)	68.5 (−22.8%)	33.2 (−16.4%)	14.2 (−31.5%)	11.2 (−37.0%)	6.6 (−42.6%)	1.7 (−45.0%)
*d60*	15.7 (−3.0%)	65.7 (−26.1%)	30.1 (−24.2%)	14.0 (−32.6%)	12.9 (−27.4%)	7.6 (−34.5%)	1.7 (−45.7%)
Jukkoku*_sd1* + *d60*	15.8 (−2.2%)	48.6 (−45.3%)	26.7 (−32.7%)	10.7 (−48.5%)	7.9 (−55.8%)	3.1 (−72.8%)	0.5 (−83.5%)
IR8*_sd1* + *d60*	15.7 (−3.3%)	50.2 (−43.5%)	30.1 (−24.3%)	11.2 (−46.0%)	6.6 (−63.0%)	1.9 (−83.6%)	0.3 (−91.8%)

## Supplementary Materials

Supplementary Materials DNA sequences of* Sd1/sd1* locus.
