# Supplementary material for: Genetic Performance of the Semidwarfing Allele sd1 Derived from a Japonica Rice Cultivar and Minimum Requirements to Detect Its Single-Nucleotide Polymorphism by MiSeq Whole-Genome Sequencing
Source: Biomed Res Int. 2018 Apr 3;2018:4241725. doi: 10.1155/2018/4241725 (PMC5903320; doi:10.1155/2018/4241725)
Supplement: Supplementary Materials — DNA sequences of Sd1/sd1 locus. [file 4241725.f1.pdf]

Supplementary file of DNA sequences of *Sd1*/*sd1* locus

>Koshihikari_*Sd1*
TGCCCAGACAGCTCGCCCTGCACACACACACACACTCACACTCACACACGCTCTCAACTCACTCCCGCTC
AACACAGCGCTCACTTCTCATCTCCAATCTCATGGTGGCCGAGCACCCCACGCCACCACAGCCGCACCAA
CCACCGCCCATGGACTCCACCGCCGGCTCTGGCATTGCCGCCCCGGCGGCGGCGGCGGTGTGCGACCTGA
GGATGGAGCCCAAGATCCCGGAGCCATTCGTGTGGCCGAACGGCGACGCGAGGCCGGCGTCGGCGGCGGA
GCTGGACATGCCCGTGGTCGACGTGGGCGTGCTCCGCGACGGCGACGCCGAGGGGCTGCGCCGCGCCGCG
GCGCAGGTGGCCGCCGCGTGCGCCACGCACGGGTTCTTCCAGGTGTCCGAGCACGGCGTCGACGCCGCTC
TGGCGCGCGCCGCGCTCGACGGCGCCAGCGACTTCTTCCGCCTCCCGCTCGCCGAGAAGCGCCGCGCGCG
CCGCGTCCCGGGCACCGTGTCCGGCTACACCAGCGCCCACGCCGACCGCTTCGCCTCCAAGCTCCCATGG
AAGGAGACCCTCTCCTTCGGCTTCCACGACCGCGCCGCCGCCCCCGTCGTCGCCGACTACTTCTCCAGCA
CCCTCGGCCCCGACTTCGCGCCAATGGGGTAATTAAAACGATGGTGGACGACATTGCATTTCAAATTCAA
AACAAATTCAAAACACACCGACCGAGATTATGCTGAATTCAAACGCGTTTGTGCGCGCAGGAGGGTGTAC
CAGAAGTACTGCGAGGAGATGAAGGAGCTGTCGCTGACGATCATGGAACTCCTGGAGCTGAGCCTGGGCG
TGGAGCGAGGCTACTACAGGGAGTTCTTCGCGGACAGCAGCTCAATCATGCGGTGCAACTACTACCCGCC
ATGCCCGGAGCCGGAGCGGACGCTCGGCACGGGCCCGCACTGCGACCCCACCGCCCTCACCATCCTCCTC
CAGGACGACGTCGGCGGCCTCGAGGTCCTCGTCGACGGCGAATGGCGCCCCGTCAGCCCCGTCCCCGGCG
CCATGGTCATCAACATCGGCGACACCTTCATGGTAAACCATCTCCTATTCTCCTCTCCTCTGTTCTCCTC
TGCTTCGAAGCAACAGAACAAGTAATTCAAGCTTTTTTTTCTCTCTCGCGCGAAATTGACGAGAAAAATA
AGATCGTGGTAGGGGCGGGGCTTTCAGCTGAAAGCGGGAAGAAACCGACCTGACGTGATTTCTCTGTTCC
AATCACAAACAATGGAATGCCCCACTCCTCCATGTGTTATGATTTATCTCACATCTTATAGTTAATAGGA
GTAAGTAACAAGCTACTTTTTTCATATTATAGTTCGTTTGATTTTTTTTTTTTTTAAGTTTTTTTAGTTTT
ATCCAAATTTATTGAAAAACTTAGCAACGTTTATAATACCAAATTAGTCTCATTTAGTTTAATATTGTAT
ATATTTTGATAATATATTTATGTTATATTAAAAATATTACTATATTTTTCTATAAACATTATTAAAAGCC
ATTTATAATATAAAATGGAAGGAGTAATTAATATGGATCTCCCCCGACATGAGAATATTTTCCGATGGTG
TGACGACGCCATGTAAGCTTCGGTGGGCCTGGACGGCCAGAGGTGCCAACAGCCACGTCCAACAACCCCT
GGGTCCCCCCCTAACACTCCAAACAGTAGTGAGTAGTGTCTCGTCGCGTTTTAGTATTTGATGACAAACA
AAGTGTGAGTTGAGTTAGCCACCACCAACTTGCACACGAGCACATACATTTGTGTCCATTCTCGCCAGTC
ACTTCCATCTCTAGTCCTAACTCCTATCTAGCGATGTAAGCGGATAATTTCATCATCCGTATATAAACCT
GTTTGTTATAGTTAATTTCCTATATAATACTATAACAGTATACATTTTAAAAGAAAACAAAATTAGGATA
AACAGGCCCTGCTCCTATCCATCCATGGCACTTGGAAGGACCAGACTCGGTCATGCCATGCCAAGCCAAG
ATATGGGTTATGGAAGAGTAGAGAAGAGGAGAGATGAGAGATAAGCATGCGTTCTCCTCCTCGTTGGATG
TGTATTTTGGAGGGATTTGTGTAGTAGTAGCAGCGGCGCCGCGGGGACGGATGCGGATGGTGGCGCTTTC
GGTGGCGTTTTCCCGGGGGGGTTTTGGTTTGGCGCTTGGGGGGGATGGCATGGCGCGGCGTGCGGCTGCA
CGCCACACACACGCGCGCGCACGCACGTACGTCGTCGTCGCCGCGGGCGGACGGTAGCTTAGGGTGGTGT

GTTCCGCGCGCGGGCGCGGATTGTTCCATGCCGATCGATTTGGCGCCACCCTCGCCGCGGCTCTTGTCGC
GTCGTGCGCCTCTCTCGCGCGGTTTGTCCTTGTCGCGTTGCTCAGCCGGCGACGGGGGCACGGACATTGG
CGATGTAGCCCTGCACGTGTCGGCCTCTCCGTTGATGAATGATGATGTATGTATGTATTTTTTTTTGTCT
GAAGGAATTTGTGGGGAATTGTTGTGTGTGCAGGCGCTGTCGAACGGGAGGTATAAGAGCTGCCTGCACA
GGGCGGTGGTGAACCAGCGGCGGGAGCGGCGGTCGCTGGCGTTCTTCCTGTGCCCGCGGGAGGACAGGGT
GGTGCGGCCGCCGCCGAGCGCCGCCACGCCGCAGCACTACCCGGACTTCACCTGGGCCGACCTCATGCGC
TTCACGCAGCGCCACTACCGCGCCGACACCCGCACGCTCGACGCCTTCACGCGCTGGCTCGCGCCGCCGG
CCGCCGACGCCGCCGCGACGGCGCAGGTCGAGGCGGCCAGCTGATCGCCGAACGGAACGAAACGGAACGA
ACAGAAGCCGATTTTTGGCGGGGCCCACGCCCACGTGAGGCCCCACGTGGACAGTGGGCCCGGGCGGAGG
TGGCACCCACGTGGACCGCGGGCCCCGCGCCGCCTTCCAATTTTGGACCCTACCGCTGTACATATTCATA
TATTGCAAGAAGAAGCAAAACGTACGTGTGGGTTGGGTTGGGCTTCTCTCTATTACTAAAAAAAATATAA
TGGAACGACGGATGAATGGATGCTTATTTATTTATCTAAATTGAATTCGAATTCGGCTCA

>Koshihikari Jukkoku_*sd1*
TGCCCAGACAGCTCGCCCTGCACACACACACACACTCACACTCACACACGCTCTCAACTCACTCCCGCTC
AACACAGCGCTCACTTCTCATCTCCAATCTCATGGTGGCCGAGCACCCCACGCCACCACAGCCGCACCAA
CCACCGCCCATGGACTCCACCGCCGGCTCTGGCATTGCCGCCCCGGCGGCGGCGGCGGTGTGCGACCTGA
GGATGGAGCCCAAGATCCCGGAGCCATTCGTGTGGCCGAACGGCGACGCGAGGCCGGCGTCGGCGGCGGA
GCTGGACATGCCCGTGGTCGACGTGGGCGTGCTCCGCGACGGCGACGCCGAGGGGCTGCGCCGCGCCGCG
GCGCAGGTGGCCGCCGCGTGCGCCACGCACGTGTTCTTCCAGGTGTCCGAGCACGGCGTCGACGCCGCTC
TGGCGCGCGCCGCGCTCGACGGCGCCAGCGACTTCTTCCGCCTCCCGCTCGCCGAGAAGCGCCGCGCGCG
CCGCGTCCCGGGCACCGTGTCCGGCTACACCAGCGCCCACGCCGACCGCTTCGCCTCCAAGCTCCCATGG
AAGGAGACCCTCTCCTTCGGCTTCCACGACCGCGCCGCCGCCCCCGTCGTCGCCGACTACTTCTCCAGCA
CCCTCGGCCCCGACTTCGCGCCAATGGGGTAATTAAAACGATGGTGGACGACATTGCATTTCAAATTCAA
AACAAATTCAAAACACACCGACCGAGATTATGCTGAATTCAAACGCGTTTGTGCGCGCAGGAGGGTGTAC
CAGAAGTACTGCGAGGAGATGAAGGAGCTGTCGCTGACGATCATGGAACTCCTGGAGCTGAGCCTGGGCG
TGGAGCGAGGCTACTACAGGGAGTTCTTCGCGGACAGCAGCTCAATCATGCGGTGCAACTACTACCCGCC
ATGCCCGGAGCCGGAGCGGACGCTCGGCACGGGCCCGCACTGCGACCCCACCGCCCTCACCATCCTCCTC
CAGGACGACGTCGGCGGCCTCGAGGTCCTCGTCGACGGCGAATGGCGCCCCGTCAGCCCCGTCCCCGGCG
CCATGGTCATCAACATCGGCGACACCTTCATGGTAAACCATCTCCTATTCTCCTCTCCTCTGTTCTCCTC
TGCTTCGAAGCAACAGAACAAGTAATTCAAGCTTTTTTTTCTCTCTCGCGCGAAATTGACGAGAAAAATA
AGATCGTGGTAGGGGCGGGGCTTTCAGCTGAAAGCGGGAAGAAACCGACCTGACGTGATTTCTCTGTTCC
AATCACAAACAATGGAATGCCCCACTCCTCCATGTGTTATGATTTATCTCACATCTTATAGTTAATAGGA
GTAAGTAACAAGCTACTTTTTTCATATTATAGTTCGTTTGATTTTTTTTTTTTTTAAGTTTTTTTAGTTTT
ATCCAAATTTATTGAAAAACTTAGCAACGTTTATAATACCAAATTAGTCTCATTTAGTTTAATATTGTAT
ATATTTTGATAATATATTTATGTTATATTAAAAATATTACTATATTTTTCTATAAACATTATTAAAAGCC

ATTTATAATATAAAATGGAAGGAGTAATTAATATGGATCTCCCCCGACATGAGAATATTTTCCGATGGTG
TGACGACGCCATGTAAGCTTCGGTGGGCCTGGACGGCCAGAGGTGCCAACAGCCACGTCCAACAACCCCT
GGGTCCCCCCCTAACACTCCAAACAGTAGTGAGTAGTGTCTCGTCGCGTTTTAGTATTTGATGACAAACA
AAGTGTGAGTTGAGTTAGCCACCACCAACTTGCACACGAGCACATACATTTGTGTCCATTCTCGCCAGTC
ACTTCCATCTCTAGTCCTAACTCCTATCTAGCGATGTAAGCGGATAATTTCATCATCCGTATATAAACCT
GTTTGTTATAGTTAATTTCCTATATAATACTATAACAGTATACATTTTAAAAGAAAACAAAATTAGGATA
AACAGGCCCTGCTCCTATCCATCCATGGCACTTGGAAGGACCAGACTCGGTCATGCCATGCCAAGCCAAG
ATATGGGTTATGGAAGAGTAGAGAAGAGGAGAGATGAGAGATAAGCATGCGTTCTCCTCCTCGTTGGATG
TGTATTTTGGAGGGATTTGTGTAGTAGTAGCAGCGGCGCCGCGGGGACGGATGCGGATGGTGGCGCTTTC
GGTGGCGTTTTCCCGGGGGGGTTTTGGTTTGGCGCTTGGGGGGGATGGCATGGCGCGGCGTGCGGCTGCA
CGCCACACACACGCGCGCGCACGCACGTACGTCGTCGTCGCCGCGGGCGGACGGTAGCTTAGGGTGGTGT
GTTCCGCGCGCGGGCGCGGATTGTTCCATGCCGATCGATTTGGCGCCACCCTCGCCGCGGCTCTTGTCGC
GTCGTGCGCCTCTCTCGCGCGGTTTGTCCTTGTCGCGTTGCTCAGCCGGCGACGGGGGCACGGACATTGG
CGATGTAGCCCTGCACGTGTCGGCCTCTCCGTTGATGAATGATGATGTATGTATGTATTTTTTTTTGTCT
GAAGGAATTTGTGGGGAATTGTTGTGTGTGCAGGCGCTGTCGAACGGGAGGTATAAGAGCTGCCTGCACA
GGGCGGTGGTGAACCAGCGGCGGGAGCGGCGGTCGCTGGCGTTCTTCCTGTGCCCGCGGGAGGACAGGGT
GGTGCGGCCGCCGCCGAGCGCCGCCACGCCGCAGCACTACCCGGACTTCACCTGGGCCGACCTCATGCGC
TTCACGCAGCGCCACTACCGCGCCGACACCCGCACGCTCGACGCCTTCACGCGCTGGCTCGCGCCGCCGG
CCGCCGACGCCGCCGCGACGGCGCAGGTCGAGGCGGCCAGCTGATCGCCGAACGGAACGAAACGGAACGA
ACAGAAGCCGATTTTTGGCGGGGCCCACGCCCACGTGAGGCCCCACGTGGACAGTGGGCCCGGGCGGAGG
TGGCACCCACGTGGACCGCGGGCCCCGCGCCGCCTTCCAATTTTGGACCCTACCGCTGTACATATTCATA
TATTGCAAGAAGAAGCAAAACGTACGTGTGGGTTGGGTTGGGCTTCTCTCTATTACTAAAAAAAATATAA
TGGAACGACGGATGAATGGATGCTTATTTATTTATCTAAATTGAATTCGAATTCGGCTCA

>Koshihikari_*d60*
TGCCCAGACAGCTCGCCCTGCACACACACACACACTCACACTCACACACGCTCTCAACTCACTCCCGCTC
AACACAGCGCTCACTTCTCATCTCCAATCTCATGGTGGCCGAGCACCCCACGCCACCACAGCCGCACCAA
CCACCGCCCATGGACTCCACCGCCGGCTCTGGCATTGCCGCCCCGGCGGCGGCGGCGGTGTGCGACCTGA
GGATGGAGCCCAAGATCCCGGAGCCATTCGTGTGGCCGAACGGCGACGCGAGGCCGGCGTCGGCGGCGGA
GCTGGACATGCCCGTGGTCGACGTGGGCGTGCTCCGCGACGGCGACGCCGAGGGGCTGCGCCGCGCCGCG
GCGCAGGTGGCCGCCGCGTGCGCCACGCACGGGTTCTTCCAGGTGTCCGAGCACGGCGTCGACGCCGCTC
TGGCGCGCGCCGCGCTCGACGGCGCCAGCGACTTCTTCCGCCTCCCGCTCGCCGAGAAGCGCCGCGCGCG
CCGCGTCCCGGGCACCGTGTCCGGCTACACCAGCGCCCACGCCGACCGCTTCGCCTCCAAGCTCCCATGG
AAGGAGACCCTCTCCTTCGGCTTCCACGACCGCGCCGCCGCCCCCGTCGTCGCCGACTACTTCTCCAGCA
CCCTCGGCCCCGACTTCGCGCCAATGGGGTAATTAAAACGATGGTGGACGACATTGCATTTCAAATTCAA
AACAAATTCAAAACACACCGACCGAGATTATGCTGAATTCAAACGCGTTTGTGCGCGCAGGAGGGTGTAC

CAGAAGTACTGCGAGGAGATGAAGGAGCTGTCGCTGACGATCATGGAACTCCTGGAGCTGAGCCTGGGCG
TGGAGCGAGGCTACTACAGGGAGTTCTTCGCGGACAGCAGCTCAATCATGCGGTGCAACTACTACCCGCC
ATGCCCGGAGCCGGAGCGGACGCTCGGCACGGGCCCGCACTGCGACCCCACCGCCCTCACCATCCTCCTC
CAGGACGACGTCGGCGGCCTCGAGGTCCTCGTCGACGGCGAATGGCGCCCCGTCAGCCCCGTCCCCGGCG
CCATGGTCATCAACATCGGCGACACCTTCATGGTAAACCATCTCCTATTCTCCTCTCCTCTGTTCTCCTC
TGCTTCGAAGCAACAGAACAAGTAATTCAAGCTTTTTTTTCTCTCTCGCGCGAAATTGACGAGAAAAATA
AGATCGTGGTAGGGGCGGGGCTTTCAGCTGAAAGCGGGAAGAAACCGACCTGACGTGATTTCTCTGTTCC
AATCACAAACAATGGAATGCCCCACTCCTCCATGTGTTATGATTTATCTCACATCTTATAGTTAATAGGA
GTAAGTAACAAGCTACTTTTTTCATATTATAGTTCGTTTGATTTTTTTTTTTTTAAGTTTTTTTAGTTTT
ATCCAAATTTATTGAAAAACTTAGCAACGTTTATAATACCAAATTAGTCTCATTTAGTTTAATATTGTAT
ATATTTTGATAATATATTTATGTTATATTAAAAATATTACTATATTTTTCTATAAACATTATTAAAAGCC
ATTTATAATATAAAATGGAAGGAGTAATTAATATGGATCTCCCCCGACATGAGAATATTTTCCGATGGTG
TGACGACGCCATGTAAGCTTCGGTGGGCCTGGACGGCCAGAGGTGCCAACAGCCACGTCCAACAACCCCT
GGGTCCCCCCCTAACACTCCAAACAGTAGTGAGTAGTGTCTCGTCGCGTTTTAGTATTTGATGACAAACA
AAGTGTGAGTTGAGTTAGCCACCACCAACTTGCACACGAGCACATACATTTGTGTCCATTCTCGCCAGTC
ACTTCCATCTCTAGTCCTAACTCCTATCTAGCGATGTAAGCGGATAATTTCATCATCCGTATATAAACCT
GTTTGTTATAGTTAATTTCCTATATAATACTATAACAGTATACATTTTAAAAGAAAACAAAATTAGGATA
AACAGGCCCTGCTCCTATCCATCCATGGCACTTGGAAGGACCAGACTCGGTCATGCCATGCCAAGCCAAG
ATATGGGTTATGGAAGAGTAGAGAAGAGGAGAGATGAGAGATAAGCATGCGTTCTCCTCCTCGTTGGATG
TGTATTTTGGAGGGATTTGTGTAGTAGTAGCAGCGGCGCCGCGGGGACGGATGCGGATGGTGGCGCTTTC
GGTGGCGTTTTCCCGGGGGGGTTTTGGTTTGGCGCTTGGGGGGGATGGCATGGCGCGGCGTGCGGCTGCA
CGCCACACACACGCGCGCGCACGCACGTACGTCGTCGTCGCCGCGGGCGGACGGTAGCTTAGGGTGGTGT
GTTCCGCGCGCGGGCGCGGATTGTTCCATGCCGATCGATTTGGCGCCACCCTCGCCGCGGCTCTTGTCGC
GTCGTGCGCCTCTCTCGCGCGGTTTGTCCTTGTCGCGTTGCTCAGCCGGCGACGGGGGCACGGACATTGG
CGATGTAGCCCTGCACGTGTCGGCCTCTCCGTTGATGAATGATGATGTATGTATGTATTTTTTTTTGTCT
GAAGGAATTTGTGGGGAATTGTTGTGTGTGCAGGCGCTGTCGAACGGGAGGTATAAGAGCTGCCTGCACA
GGGCGGTGGTGAACCAGCGGCGGGAGCGGCGGTCGCTGGCGTTCTTCCTGTGCCCGCGGGAGGACAGGGT
GGTGCGGCCGCCGCCGAGCGCCGCCACGCCGCAGCACTACCCGGACTTCACCTGGGCCGACCTCATGCGC
TTCACGCAGCGCCACTACCGCGCCGACACCCGCACGCTCGACGCCTTCACGCGCTGGCTCGCGCCGCCGG
CCGCCGACGCCGCCGCGACGGCGCAGGTCGAGGCGGCCAGCTGATCGCCGAACGGAACGAAACGGAACGA
ACAGAAGCCGATTTTTGGCGGGGCCCACGCCCACGTGAGGCCCCACGTGGACAGTGGGCCCGGGCGGAGG
TGGCACCCACGTGGACCGCGGGCCCCGCGCCGCCTTCCAATTTTGGACCCTACCGCTGTACATATTCATA
TATTGCAAGAAGAAGCAAAACGTACGTGTGGGTTGGGTTGGGCTTCTCTCTATTACTAAAAAAAATATAA
TGGAACGACGGATGAATGGATGCTTATTTATTTATCTAAATTGAATTCGAATTCGGCTCA

>Koshihikari_*d60*+*sd1*

TGCCCAGACAGCTCGCCCTGCACACACACACACACTCACACTCACACACGCTCTCAACTCACTCCCGCTC
AACACAGCGCTCACTTCTCATCTCCAATCTCATGGTGGCCGAGCACCCCACGCCACCACAGCCGCACCAA
CCACCGCCCATGGACTCCACCGCCGGCTCTGGCATTGCCGCCCCGGCGGCGGCGGCGGTGTGCGACCTGA
GGATGGAGCCCAAGATCCCGGAGCCATTCGTGTGGCCGAACGGCGACGCGAGGCCGGCGTCGGCGGCGGA
GCTGGACATGCCCGTGGTCGACGTGGGCGTGCTCCGCGACGGCGACGCCGAGGGGCTGCGCCGCGCCGCG
GCGCAGGTGGCCGCCGCGTGCGCCACGCACGTGTTCTTCCAGGTGTCCGAGCACGGCGTCGACGCCGCTC
TGGCGCGCGCCGCGCTCGACGGCGCCAGCGACTTCTTCCGCCTCCCGCTCGCCGAGAAGCGCCGCGCGCG
CCGCGTCCCGGGCACCGTGTCCGGCTACACCAGCGCCCACGCCGACCGCTTCGCCTCCAAGCTCCCATGG
AAGGAGACCCTCTCCTTCGGCTTCCACGACCGCGCCGCCGCCCCCGTCGTCGCCGACTACTTCTCCAGCA
CCCTCGGCCCCGACTTCGCGCCAATGGGGTAATTAAAACGATGGTGGACGACATTGCATTTCAAATTCAA
AACAAATTCAAAACACACCGACCGAGATTATGCTGAATTCAAACGCGTTTGTGCGCGCAGGAGGGTGTAC
CAGAAGTACTGCGAGGAGATGAAGGAGCTGTCGCTGACGATCATGGAACTCCTGGAGCTGAGCCTGGGCG
TGGAGCGAGGCTACTACAGGGAGTTCTTCGCGGACAGCAGCTCAATCATGCGGTGCAACTACTACCCGCC
ATGCCCGGAGCCGGAGCGGACGCTCGGCACGGGCCCGCACTGCGACCCCACCGCCCTCACCATCCTCCTC
CAGGACGACGTCGGCGGCCTCGAGGTCCTCGTCGACGGCGAATGGCGCCCCGTCAGCCCCGTCCCCGGCG
CCATGGTCATCAACATCGGCGACACCTTCATGGTAAACCATCTCCTATTCTCCTCTCCTCTGTTCTCCTC
TGCTTCGAAGCAACAGAACAAGTAATTCAAGCTTTTTTTTCTCTCTCGCGCGAAATTGACGAGAAAAATA
AGATCGTGGTAGGGGCGGGGCTTTCAGCTGAAAGCGGGAAGAAACCGACCTGACGTGATTTCTCTGTTCC
AATCACAAACAATGGAATGCCCCACTCCTCCATGTGTTATGATTTATCTCACATCTTATAGTTAATAGGA
GTAAGTAACAAGCTACTTTTTTCATATTATAGTTCGTTTGATTTTTTTTTTTTTAAGTTTTTTTAGTTTT
ATCCAAATTTATTGAAAAACTTAGCAACGTTTATAATACCAAATTAGTCTCATTTAGTTTAATATTGTAT
ATATTTTGATAATATATTTATGTTATATTAAAAATATTACTATATTTTTCTATAAACATTATTAAAAGCC
ATTTATAATATAAAATGGAAGGAGTAATTAATATGGATCTCCCCCGACATGAGAATATTTTCCGATGGTG
TGACGACGCCATGTAAGCTTCGGTGGGCCTGGACGGCCAGAGGTGCCAACAGCCACGTCCAACAACCCCT
GGGTCCCCCCCTAACACTCCAAACAGTAGTGAGTAGTGTCTCGTCGCGTTTTAGTATTTGATGACAAACA
AAGTGTGAGTTGAGTTAGCCACCACCAACTTGCACACGAGCACATACATTTGTGTCCATTCTCGCCAGTC
ACTTCCATCTCTAGTCCTAACTCCTATCTAGCGATGTAAGCGGATAATTTCATCATCCGTATATAAACCT
GTTTGTTATAGTTAATTTCCTATATAATACTATAACAGTATACATTTTAAAAGAAAACAAAATTAGGATA
AACAGGCCCTGCTCCTATCCATCCATGGCACTTGGAAGGACCAGACTCGGTCATGCCATGCCAAGCCAAG
ATATGGGTTATGGAAGAGTAGAGAAGAGGAGAGATGAGAGATAAGCATGCGTTCTCCTCCTCGTTGGATG
TGTATTTTGGAGGGATTTGTGTAGTAGTAGCAGCGGCGCCGCGGGGACGGATGCGGATGGTGGCGCTTTC
GGTGGCGTTTTCCCGGGGGGGTTTTGGTTTGGCGCTTGGGGGGGATGGCATGGCGCGGCGTGCGGCTGCA
CGCCACACACACGCGCGCGCACGCACGTACGTCGTCGTCGCCGCGGGCGGACGGTAGCTTAGGGTGGTGT
GTTCCGCGCGCGGGCGCGGATTGTTCCATGCCGATCGATTTGGCGCCACCCTCGCCGCGGCTCTTGTCGC
GTCGTGCGCCTCTCTCGCGCGGTTTGTCCTTGTCGCGTTGCTCAGCCGGCGACGGGGGCACGGACATTGG
CGATGTAGCCCTGCACGTGTCGGCCTCTCCGTTGATGAATGATGATGTATGTATGTATTTTTTTTTGTCT

GAAGGAATTTGTGGGGAATTGTTGTGTGTGCAGGCGCTGTCGAACGGGAGGTATAAGAGCTGCCTGCACA
GGGCGGTGGTGAACCAGCGGCGGGAGCGGCGGTCGCTGGCGTTCTTCCTGTGCCCGCGGGAGGACAGGGT
GGTGCGGCCGCCGCCGAGCGCCGCCACGCCGCAGCACTACCCGGACTTCACCTGGGCCGACCTCATGCGC
TTCACGCAGCGCCACTACCGCGCCGACACCCGCACGCTCGACGCCTTCACGCGCTGGCTCGCGCCGCCGG
CCGCCGACGCCGCCGCGACGGCGCAGGTCGAGGCGGCCAGCTGATCGCCGAACGGAACGAAACGGAACGA
ACAGAAGCCGATTTTTGGCGGGGCCCACGCCCACGTGAGGCCCCACGTGGACAGTGGGCCCGGGCGGAGG
TGGCACCCACGTGGACCGCGGGCCCCGCGCCGCCTTCCAATTTTGGACCCTACCGCTGTACATATTCATA
TATTGCAAGAAGAAGCAAAACGTACGTGTGGGTTGGGTTGGGCTTCTCTCTATTACTAAAAAAAATATAA
TGGAACGACGGATGAATGGATGCTTATTTATTTATCTAAATTGAATTCGAATTCGGCTCA

SNP is shown as red.
